# Feasibility of Same-Day Discharge After Appendectomy in Pediatric Patients: A Systematic Review and Meta-Analysis

**DOI:** 10.3389/fped.2022.944405

**Published:** 2022-07-04

**Authors:** Yifei Zheng, Shiqin Qi

**Affiliations:** Department of General Surgery, Anhui Provincial Children’s Hospital, Hefei, China

**Keywords:** acute appendicitis, laparoscopic appendectomy, same-day discharge, children, adolescents, meta-analysis

## Abstract

**Background:**

To compare the readmission rate, rate of urgent/unplanned visits to emergency, complication rate along with cost of health care among children and adolescents who were discharged the same day following a laparoscopic appendectomy and those who were not discharged on the same day.

**Methods:**

A systematic search was performed in the PubMed, Embase, and Scopus databases. Randomized controlled trials and studies, observational in design, were considered for inclusion. The included studies were conducted in children and adolescents with uncomplicated appendicitis undergoing laparoscopic appendectomy and compared outcomes of interest between patients that were discharged the same day (SDD group) following the operation and patients that were discharged within 2 days post-operatively (non-SSD group). Statistical analysis was performed using STATA software. Effect sizes were reported as pooled odds ratio (OR) or weighted mean difference (WMD) with 95% confidence intervals.

**Results:**

A total of 13 studies with 32,021 children and adolescents were included. There was no significant difference in the risks of unplanned visit to the emergency department (OR 1.07, 95% CI: 0.78, 1.47), readmission (OR 0.83, 95% CI: 0.66, 1.05), reoperation/re-intervention (OR 1.73, 95% CI: 0.19, 16.2) and complications (OR 0.84, 95% CI: 0.67, 1.06) in both groups of patients. Patients in the SDD group had slightly lower risk of wound infection/complication (OR 0.74, 95% CI: 0.57, 0.96) compared to patients in the non-SDD group. Those with SDD had to incur comparatively lesser hospital expense (in USD) compared to those with no SDD (WMD −2587.4, 95% CI: −4628.3, −546.6).

**Conclusion:**

In children and adolescents with uncomplicated acute appendicitis undergoing laparoscopic appendectomy, same-day discharge is not associated with increased readmission risk, unplanned visits to emergency, and complications. Further, SDD is associated with lower cost of hospital care. Adoption of SDD in this subset of children and adolescents may be encouraged.

**Systematic Review Registration:**

[www.crd.york.ac.uk/prospero], identifier [CRD420 22320539].

## Background

Acute appendicitis (AA) is a progressive disease characterized by acute inflammation and infection of the appendix (1, 2). If not addressed promptly, it can lead to necrosis and perforation of the appendiceal wall (1, 2). Complicated appendicitis is defined as the presence of perforation, peri-appendicular abscess, or acute inflammation of the peritoneum (clinically called peritonitis), secondary to appendicular infection (3). It is a common surgical emergency among children and accounts for around 1–2% of all pediatric unit surgical admissions (4, 5). The global incidence of appendicitis ranges from 100 to 200 per 100,000 person-years (6).

Using data from the Nationwide Inpatient Sample in the United States, Davies et al. noted that appendicitis-related hospitalizations account for a major proportion of all childhood hospitalizations and lead to a substantial healthcare-related financial burden (7). Similarly, another study using the Dutch healthcare reimbursement registry showed that appendicitis along with appendectomy contributes to a substantial clinical and economic burden (8). While surgery remains the gold standard for management, there is a substantial risk of complications and a notable financial burden associated with appendicectomy (8–10). Some of the documented risks of appendicectomy include wound infection, post-operative ileus and intra-abdominal abscess. The rate of post-operative complications is usually lower for uncomplicated AA and laparoscopic appendicectomy compared to complicated AA and open appendectomy, respectively (11).

Laparoscopic appendectomy is the most commonly performed surgery for uncomplicated appendicitis (2, 12, 13). Studies, in both adult and pediatric populations, have examined the possibility of the same-day discharge (SDD). A recent review was conducted to assess the safety of the same-day discharge after appendectomy in adults and children with acute appendicitis (14). The review included 17 studies and performed a comparative analysis between same-day discharge and non-same day discharge. The majority of the studies included adult subjects. The review concluded that for the cases of uncomplicated appendicitis, SDD was safe and was not associated with an increased risk of readmission, unplanned visits to, the hospital, or complications (14). Recently, there has been a lot of interest in evaluating the safety of SDD in children and adolescents undergoing laparoscopic appendectomy for uncomplicated appendicitis. There is a need, therefore, to summarize the findings of numerous new studies and to present updated evidence that may be used in optimizing current clinical pediatric practice guidelines. To the best of our knowledge, there have been no prior reviews that especially focused on pediatric and adolescent populations. The primary goal of our meta-analysis is to compare the readmission rate, rate of urgent/unplanned visits to the emergency department, and complication rate in children and adolescents that were discharged the same day following a laparoscopic appendectomy and those who were not discharged on the same day. Additionally, we also examined difference in the cost of health care incurred among the two groups of subjects.

## Materials and Methods

### Search Strategy

The study protocol was registered at the International Prospective Registry of Systematic Reviews (PROSPERO; CRD42022320539). We attempted to identify studies that were carried out in children and adolescents with uncomplicated appendicitis undergoing laparoscopic appendectomy, and that compared clinical outcomes based on the timing of discharge post-operatively i.e., same-day discharge and overnight stay and/or discharge within 2 days post-operatively. Studies with discharge later than 2 days were excluded, as that would have implied post-operative complications necessitating longer hospital stay. The outcomes of interest were risk of readmission, an urgent visit to the emergency department, reoperation/re-intervention, and complications along with hospital expenses incurred.

A detailed systematic search was done using search strategy in PubMed, Embase, and Scopus databases for English language papers that were published until 15th March 2022. The search strategy used the following medical subject heading (MeSH) terminology along with free text words: (appendicitis OR uncomplicated appendicitis OR non-perforated appendicitis OR appendicectomy OR laparoscopic appendicectomy) AND (hospital discharge OR ambulatory OR outpatient OR day-case OR day surgery OR ambulatory surgery OR same-day discharge OR overnight stay) AND (children OR young children OR adolescents) AND (clinical outcomes OR readmission OR complication OR reoperation OR unplanned visit). This meta-analysis was conducted following Preferred Reporting Items for Systematic Reviews and Meta-Analyses (PRISMA) guidelines (15).

### Selection Criteria and Methods

There were pre-defined inclusion and exclusion criteria which were strictly followed during the selection of the studies. These criteria are mentioned below:

#### Inclusion Criteria

Only randomized controlled trials (RCT) or studies that were non-experimental/observational in design were considered for inclusion. We selected studies that were done on children and adolescents with uncomplicated appendicitis undergoing laparoscopic appendectomy. Uncomplicated appendicitis was considered when there was a lack of perforation, peritonitis, gangrenous changes, or formation of a peri-appendicular abscess (2, 3). Only studies that compared outcomes of interest between patients that were discharged the same day (Same day discharge, SDD group) following the operation and those that were not discharged on the same day but within 2 days post-operatively (non-SDD group).

#### Exclusion Criteria

Case reports and review articles, studies that included children with complicated appendicitis or children undergoing open appendectomy, and studies that did not compare outcomes of interest between SDD and non- SDD patients were excluded.

### Process of Study Screening

After the removal of duplicates, the list of potentially eligible studies was then screened by two independent reviewers based on the inclusion and exclusion criteria. Potentially eligible articles not reporting the relevant outcomes were excluded. The articles satisfying the inclusion and exclusion criteria were then subjected to a full-text assessment.

As an *a priori* principle, all disagreements were resolved by discussion between the two study authors. The bibliography of the included studies was screened for additional potentially relevant studies.

### Data Extraction and Quality Assessment

We used a pre-tested extraction sheet. The data extraction was done by two authors independently. The key variables were as follows: study identifier (i.e., the name of the first author and the year of publication), the design of the study and place where the study was conducted, important characteristics of the study subjects, sample size, and the key findings. The quality of the included studies was assessed using the Newcastle-Ottawa Quality Assessment Scale for observational studies. This assessment was done independently by two study authors that performed the data extraction (16).

### Statistical Analysis

The pooled effect sizes were reported as odds ratio (OR) for categorical outcomes and as weighted mean differences (WMD) for continuous outcomes. We also reported 95% confidence intervals along with the pooled odds ratio. Heterogeneity was presented using I^2^, and the random-effects model was used when I^2^ was above 40% (17). *P* < 0.05 indicated statistical significance. We used Egger’s test to assess and report publication bias (18). All the analysis were done using STATA (version 16.0).

## Results

### Selection of Articles

**FIGURE 1:**
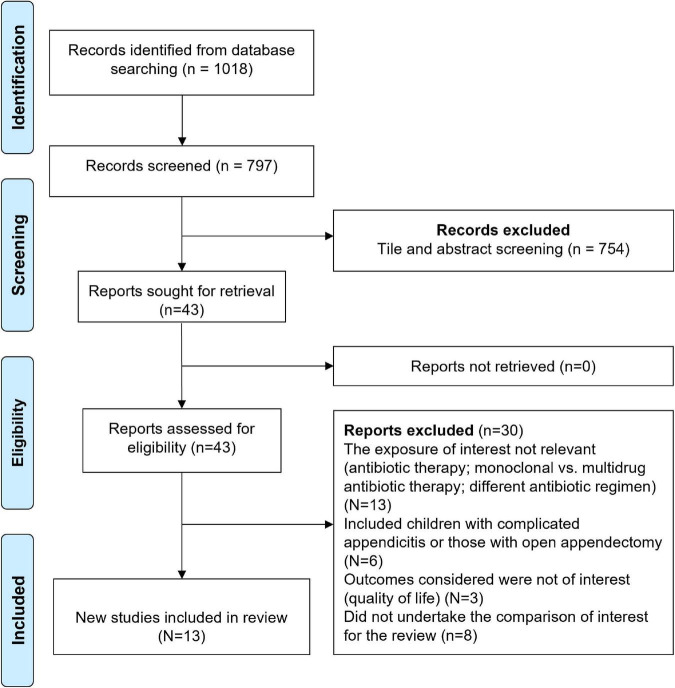
Selection process of the studies included in the review.

After the systematic search across the databases, a total of 1,018 citations were obtained. Of them, 797 relevant citations remained after excluding duplicates (Figure 1). Additional 754 citations were removed after the titles and abstracts. The full text of 43 studies was retrieved and thoroughly read. Finally, 13 studies comprising of 32,021 children and adolescents were included in the review (19–31).

### Study Characteristics, and Quality of the Included Studies

As summarized in Table 1, all the included studies were observational in design. There were four prospective cohort studies and nine studies with retrospective designs. All the studies were conducted in the United States.

**TABLE 1:** Characteristics of the studies included in the meta-analysis.

Author (year)	Study design; Country	Participant characteristics	Sample size	Key outcomes (same day discharge compared to overnight stay/admission) within 30-days post-operatively
Alkhoury et al. (19)	Prospective; United States	Children with non-perforated, uncomplicated appendicitis; Both groups had a mean age of ∼12 years; male to female ratio of 2:1 in both groups; mean surgical time similar in the 2 groups (∼23 min) Reasons for overnight stay: Hours too late for discharge (78%); medical issue (11%); social reasons (11%)	Same day discharge (SDD) (*N* = 162); overnight stay (ONS) (*N* = 45)	Wound/umbilical infection: OR 0.54 (95% CI; 0.13, 2.24) Urgent post-operative visit: OR 1.72 (95% CI; 0.37, 7.98) Complication*: OR 1.22 (95% CI; 0.33, 4.49) Readmission: OR 1.11 (95% CI; 0.12, 10.2)*umbilical wound infection
Benedict et al. (20)	Retrospective; United States	Children with non-perforated, uncomplicated appendicitis; median operative time similar [SDD-24 min; ONS-26.5 min]; 41% female in both groups; Median age of participants similar [SDD- 11; ONS-10.3] Reasons for overnight stay: Hours too late for discharge (6%); medical comorbidities (16%); pain (14%); nausea/emesis (14%); no specific reason reported (50%)	Same day discharge (SDD) (*N* = 495); overnight stay (ONS) (*N* = 74)	Readmission: OR 0.39 (95% CI; 0.10, 1.50) Complication*: OR 1.36 (95% CI; 0.07, 25.6) *includes small bowel obstruction/post-operative ileus
Cairo et al. (21)	Retrospective; United States	Children with non-perforated, uncomplicated acute appendicitis; similar proportion of males in both groups (62.6% vs. 60.9%); Mean age of participants similar [SDD- 11.2 yrs; within day 2–11.0 yrs]; proportion obese similar (30.3% vs. 29.8%); no corticosteroid use in >99% children in both groups	Same day discharge (SDD) (*N* = 4662); Day 1 or Day 2 discharge (*N* = 16319)	Readmission: OR 0.82 (95% CI; 0.51, 1.04) Wound complication: OR 0.75 (95% CI; 0.56, 1.01)
Cheng et al. (22)	Retrospective; United States	Children with non-perforated, uncomplicated acute appendicitis; similar proportion of males in both groups (60% vs. 55%); Mean age similar [SDD- 11.9 yrs; no-SDD-10.6 yrs]; proportion obese similar (5.9% vs. 7.1%); median time between consultation and operation (in hrs) was higher in non-SDD group (10.9 vs. 4.3) Median hospital stay in non-SDD group was 23.5 h	Same day discharge (SDD) (*N* = 110); no same day discharge (no-SDD) (*N* = 75)	Readmission: OR 1.02 (95% CI; 0.17, 6.27) Complication*: OR 0.22 (95% CI; 0.01, 5.60) Urgent post-operative visit: OR 0.22 (95% CI; 0.02, 2.16) Cost of hospital care (USD; mean, SD): 29195 (6900) vs. 33703 (11400) *myositis
Gee et al. (23)	Retrospective; United States	Children that underwent laparoscopic appendectomy for uncomplicated appendicitis; no significant difference in gender (around 40% females in both groups), age (majority in both groups were in the age range of 7–12 years, 57%) or race.	Same day discharge (SDD) (*N* = 382); overnight stay (ONS) (*N* = 467)	Readmission: OR 0.40 (95% CI; 0.08, 2.02) Complication*: OR 1.25 (95% CI; 0.81, 1.93) Urgent post-operative visit: OR 0.99 (95% CI; 0.56, 1.78) Wound infection: OR 0.55 (95% CI; 0.19, 1.60) Cost of hospital care (USD; mean, SD): 29150 (1105) vs. 34827 (1384) *abscess, nausea/vomiting, pain, surgical site infection
Halter et al. (24)	Retrospective; United States	Appendicitis without complications in children; mean age similar in the two groups (11.1 yrs; 10.6 yrs); proportion of females (SDD: 35%; next day: 44%)	Same day discharge (SDD) (*N* = 121); discharged next day (*N* = 115)	Readmission: OR 0.31 (95% CI; 0.03, 3.04) Complication*: OR 0.31 (95% CI; 0.03, 3.04) Urgent post-operative visit: OR 2.64 (95% CI; 0.68, 10.2) Cost of hospital care (USD; mean, SD): 10551 (2165) vs. 12691 (3507) *wound hematoma, ileus, partial small bowel obstruction
Gurien et al. (25)	Retrospective; United States	Children with uncomplicated appendicitis; no differences in age (mean age of 11.9 yrs in SDD and 10.8 yrs in other group) or gender of patients (male; around 60% in both groups). Longer hospital stays due to surgeon instructions (43%); medical issues (such as nausea/vomiting/pain) (29%); other reasons (29%)	Same day discharge (SDD) (*N* = 94); discharged after >24 h (*N* = 14)	Readmission: OR 0.47 (95% CI; 0.02, 11.9) Urgent post-operative visit: OR 2.85 (95% CI; 0.16, 52.1) No complication reported in either of the groups
Kashyap et al. (26)	Retrospective; United States	laparoscopic appendectomy for non-perforated appendicitis; no statistically significant differences in characteristics of children in both groups; mean age of 11.5 (3.6) and 11.9 (3.5) years, male/female percentages similar in both groups; similar duration of surgery in both groups (35.8 vs. 33.6 min)	Same day discharge (SDD) (*N* = 511); overnight stay (ONS) (*N* = 930)	Readmission: OR 1.14 (95% CI; 0.37, 3.50) Urgent post-operative visit: OR 0.83 (95% CI; 0.40, 1.71) Reoperation: OR 5.48 (95% CI; 0.57, 52.9) Cost of hospital care (USD; mean, SD): 32450 (3390) vs. 35420 (5430)
Wakimoto et al. (27)	Retrospective; United States	Children with uncomplicated appendicitis; similar age (median age of 11 yrs); 39% females in both groups; median BMI of 20 kg/m^2^ in both groups; 61% non-Hispanic whites in both groups	Same day discharge (SDD) (*N* = 2443); overnight stay (ONS) (*N* = 2443)	Readmission: OR 0.90 (95% CI; 0.60, 1.4) Complication*: OR 0.80 (95% CI; 0.60, 1.1) *infection/pneumonia/renal failure
Yu et al. (28)	Prospective cohort; United States	Children with uncomplicated appendicitis; Average age of 11.4 years and 64% were male. Median hospital stay in no-SDD group was 17.4 h	Same day discharge (SDD) (*N* = 185); no-SDD (*N* = 417)	Urgent post-operative visit: OR 0.71 (95% CI; 0.31, 1.60) Complication*: OR 0.51 (95% CI; 0.14, 1.82) Readmission: OR 0.22 (95% CI; 0.03, 1.74) Reoperation: OR 0.56 (95% CI; 0.06, 5.06) Cost of hospital care (USD; mean, SD): 8073 (391) vs. 8424 (420) *bleeding problem/hemoperitoneum, surgical site infection
Putnam et al. (29)	Prospective cohort; United States	Children with uncomplicated appendicitis undergoing laparoscopic surgery (>90%); sex (M:F ratio of 1.7), age (median age of 10 years), and body mass index (median of 19.0 kg/m^2^) similar between the two groups. Median hospital stay in no-SDD group was 24 h	Same day discharge (SDD) (*N* = 478); no-SDD (*N* = 316)	Urgent post-operative visit: OR 2.73 (95% CI; 1.10, 6.76) Wound infection: OR 1.74 (95% CI; 0.61, 4.93) Readmission: OR 2.87 (95% CI; 0.96, 8.63) Cost of hospital care (USD; mean, SD): 2719 (926) vs. 3090 (996)
Devin et al. (30)	Prospective cohort; United States	Children under 18 years admitted for laparoscopic appendectomy for acute, uncomplicated appendicitis; around 60% males in both groups; median age in both groups 11.3 yrs; in both groups, majority were non-Hispanics (around 81%)	Same day discharge (SDD) (*N* = 268); no-SDD (*N* = 307)	Readmission: OR 0.45 (95% CI; 0.14, 1.45) Urgent post-operative visit: OR 0.94 (95% CI; 0.45, 1.95) Wound infection: OR 0.34 (95% CI; 0.11, 1.06) Complication*: OR 0.26 (95% CI; 0.09, 0.78) *surgical site infection/need for percutaneous drainage
Aguayo et al. (31)	Retrospective; United States	Children that underwent laparoscopic appendectomy for non-perforated appendicitis; mean age similar in both groups (11.9 vs. 11.5 yrs); similar proportion of males in both groups (66% vs. 61%);	Same day discharge (SDD) (*N* = 128); overnight stay (ONS) (*N* = 460)	Urgent post-operative visit: OR 0.86 (95% CI; 0.34, 2.13) Complication: OR 0.65 (95% CI; 0.14, 2.96) Readmission: OR 0.60 (95% CI; 0.07, 5.0)

All the included studies were done in children and adolescents with non-perforated non-gangrenous uncomplicated appendicitis who underwent laparoscopic appendicectomy. The outcomes reported in the included studies were within 30 days post-operatively. There was no statistically significant difference in the baseline characteristics of the patients, such as age, gender distribution, body mass index, and ethnicity. All the studies were done on children and adolescents under 18 years of age and the mean age ranged from 10 to 12 years (Table 1). In six studies, the comparison group included children with an overnight stay. In the remaining studies, the children were discharged within two days post-operatively. Among studies that provided information on the reasons for overnight/prolonged post-operative stay, the key reasons were surgeon and family preference, hours too late for discharge, medical comorbidities such as pain, nausea, and vomiting, and socSial reasons (Table 1). Quality evaluation of the included studies is summarized in Supplementary Table 1. All included studies were of high to modest quality.

### Risk of the Unplanned Hospital Visit, Readmission, and Reoperation

**FIGURE 2:**
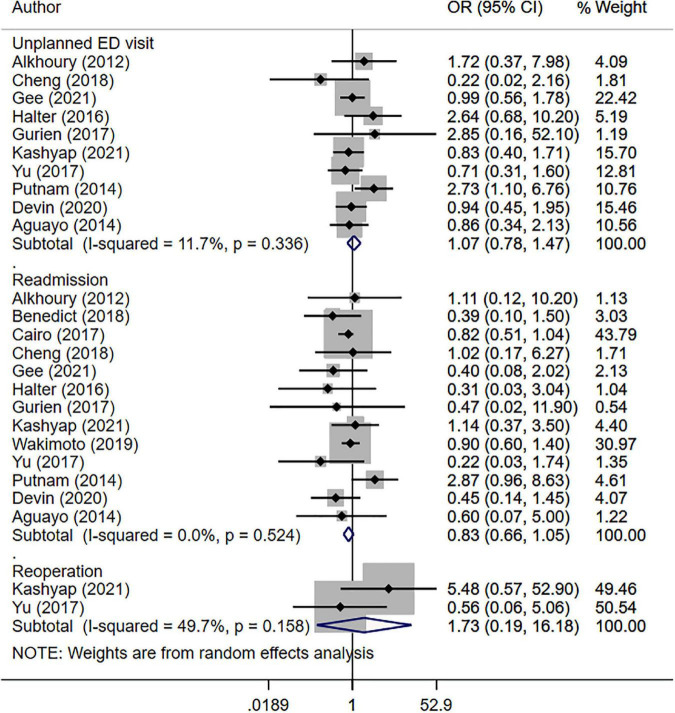
Risk of unplanned hospital visit, readmission and reoperation between those with same day discharge (SDD) and those with no SDD.

The risk of unplanned visit to the emergency department (OR 1.07, 95% CI: 0.78, 1.47; *N* = 10; I^2^ = 11.7%), readmission (OR 0.83, 95% CI: 0.66, 1.05; *N* = 13; I^2^ = 0.0%) and reoperation/re-intervention (OR 1.73, 95% CI: 0.19, 16.2; *N* = 2; I^2^ = 49.7%) was statistically similar between patients in the SDD and no-SDD groups (i.e., either overnight stay or discharge within 2 days post-operatively) (Figure 2). There was no evidence of publication bias (*P* = 0.47 for unplanned visit, *P* = 0.29 for readmission and *P* = 0.84 for reoperation).

### Risk of Complications

**FIGURE 3:**
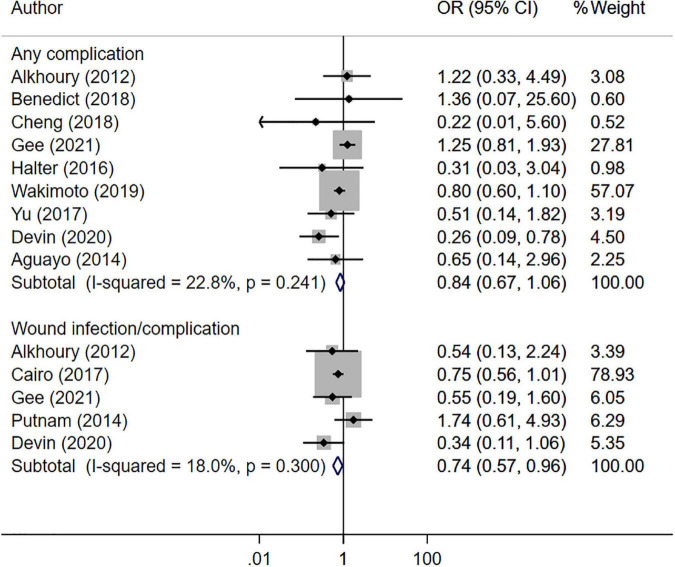
Risk of complications between those with same day discharge (SDD) and those with no SDD.

The risk of overall complications (OR 0.84, 95% CI: 0.67, 1.06; *N* = 9; I^2^ = 22.8%) was statistically similar in both SDD and non-SDD groups (Figure 3). However, in the SDD group, the risk of wound infection/complication (OR 0.74, 95% CI: 0.57, 0.96; *N* = 10; I^2^ = 11.7%) was slightly lower than in the non-SDD group. The reported complications included surgical site infection, small bowel obstruction, post-operative ileus, myositis, nausea/vomiting, abdominal pain, acute renal failure, pneumonia, wound hematoma and hemoperitoneum. There was no evidence of publication bias for the reported outcomes (*P* = 0.17 for the overall complication, *P* = 0.42 for wound infection/complication).

### Hospital Expenses Incurred

**FIGURE 4:**
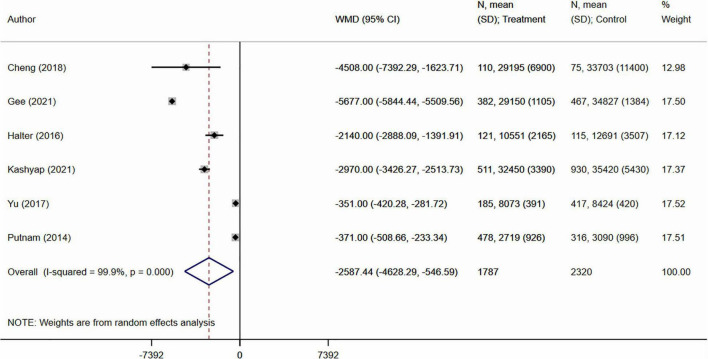
Cost of hospital care (in USD) between those with same day discharge (SDD) and those with no SDD.

Analysis suggests that those with SDD had lesser expense (in USD), compared to non-SDD subjects (WMD −2587.4, 95% CI: −4628.3, −546.6; *N* = 6; I^2^ = 99.9%) (Figure 4). Gurien et al. in their study noted that for each patient, the hospital charges for admission <24-h and >24-h were 1007 and 2237 USD more compared to those who were discharged home directly from the post-anesthesia care unit.

## Discussion

The main goal of this meta-analysis was to provide the most updated evidence on the comparative risk of readmission, re-intervention, unplanned visit to the emergency department, and complications within 30-days postoperatively in children and adolescents undergoing laparoscopic appendectomy for uncomplicated appendicitis and discharged on the same day or within 2 days post-operatively. Different time of post-operative discharge was not associated with the statistically significant difference in the risk of these outcomes in both groups of children. Patients in the same-day discharge group, however, had a lower risk of wound-related complications. It might be that same day discharge is possible because of this lower risk and those with wound infection/complication would have required additional management and/or observation, thereby necessitating stay at the hospital. These findings are in agreement with a previous review by Elisabeth et al. (14) that looked at studies done in both adult and pediatric populations and indicated that same-day discharge after laparoscopic appendectomy in patients with uncomplicated appendicitis is relatively safe and is not associated with a higher risk of readmission, complications, or unplanned hospital visits. One of the major drawbacks of the earlier review was that it did not provide stratified findings based on adult and pediatric subjects. Our review, in contrast, provided updated new data and specifically focused on the pediatric and adolescent population.

Before further discussing the findings of this meta-analysis, it is imperative to acknowledge the limitations of the analysis and draw conclusions based on these limitations. All the included studies were observational in design. Therefore, we cannot rule out the possibility that for some important confounders data were not available or not adjusted for, and there is also a risk of the selective reporting bias. Additionally, all the included studies were done in the United States which limits the external generalizability of the findings. In some of the included studies, patients with the same day discharge were compared with non-matched patients with an overnight stay, and the reasons for the overnight stay were not necessarily related to medical comorbidities e.g., too late for discharge, social reasons, etc. This may make our comparisons and the resulting findings not as reliable from a clinical perspective. Also, it should be noted that the included studies had patients that underwent laparoscopic appendectomy. Therefore, the findings of this meta-analysis will not apply to children and adolescents undergoing an open appendectomy. An important limitation is that the review did not consider other important aspect of patient satisfaction. This is a vital attribute and could impact the clinical guideline development.

Previous studies showed that appendectomies account for a substantial proportion of pediatric health care expenses and health infrastructure utilization (7–10). Our meta-analysis noted a comparatively lesser cost of hospital care (around 2600 USD lower) among those with SDD.

With an increase in the cost of health care in recent years, there has been a push toward outpatient surgery with the intent to decrease the length of hospital stay and other related costs. Global estimates suggest that the incidence of appendicitis/appendectomy is high (around 100 to 200 per 100,000 person-years) and the cost per appendectomy is also substantial (6, 8–10). Therefore, a significant reduction in health care expense due to SDD along with no increased readmission risk, unplanned visits to emergency, and other complications may provide more impetus for its adoption in routine clinical care. The findings necessitate the need to adjust the hospital protocols to ensure that appendectomies are conducted in the early part of the day so that discharges could be made on the same day. This is important as in some of the studies included in this review, one of the major reasons for an overnight stay was that it was too late in the night for the patient to be discharged (19, 20). Another possible reason for longer post-operative stay, particularly in settings with limited access to health care, could be increased distance from health care facility. Families may consider staying at the health facility so that any unforeseen complication is managed without delay. To institute a mechanism to ensure same-day discharge for children and adolescents with uncomplicated appendicitis and undergoing laparoscopic appendectomy, it is important that the discharge protocol specifies a detailed set of eligibility criteria that could be objectively assessed by the hospital staff. Furthermore, the family and caregivers of the patients should be well-informed about the signs and symptoms of possible complications and when to report back to the hospital.

While same-day discharge could be safe, it may still be very challenging to implement in a clinical setup. Dedicated investments in the education of the health care providers about the safety of this post-operative strategy as well as managing the expectations and concerns of the family members are critical for the success. Some of the ways to improve acceptance among caregivers could be promotion of post-discharge patient engagement strategies such as setting up of dedicated “hotline” for addressing parental queries, developing fast-track readmission policy, and providing transport facilities to those that are far from the health facility or during late night. While observational (mostly retrospective) studies were able to provide initial evidence on the feasibility of SDD, these findings should be confirmed either through prospective studies or randomized controlled trials. One such prospective randomized trial was conducted by Mario et al. to investigate if the implementation of the “Enhanced Recovery After Surgery (ERAS)” protocol in patients with uncomplicated acute appendicitis decreased the length of hospital stay (LOS), and, thus, allows ambulatory laparoscopic appendectomy (defined as LOS less than 12 h) (32). Using a sample size of 108 patients, the authors showed that, compared to the conventional care, ERAS implementation was associated with a significantly shorter LOS, thereby, allowing for the ambulatory management of patients. Future trials are needed to test the best possible strategies to educate health care practitioners and caregivers on the benefits of SDD and to increase the rates of adoption of this approach.

## Data Availability Statement

Publicly available datasets were analyzed in this study. A detailed systematic search was done using search strategy in PubMed, Embase, and Scopus databases for English language articles that were published until 15th March 2022.

## Author Contributions

YZ and SQ conceived and designed the study, collected the data, and performed the analysis. SQ was involved in the writing of the manuscript and responsible for the integrity of the study. Both authors have read and approved the final manuscript.

## Conflict of Interest

The authors declare that the research was conducted in the absence of any commercial or financial relationships that could be construed as a potential conflict of interest.

## Publisher’s Note

All claims expressed in this article are solely those of the authors and do not necessarily represent those of their affiliated organizations, or those of the publisher, the editors and the reviewers. Any product that may be evaluated in this article, or claim that may be made by its manufacturer, is not guaranteed or endorsed by the publisher.
