# Supplementary material for: Feasibility of Same-Day Discharge After Appendectomy in Pediatric Patients: A Systematic Review and Meta-Analysis
Source: Front Pediatr. 2022 Jul 4;10:944405. doi: 10.3389/fped.2022.944405 (PMC9289135; doi:10.3389/fped.2022.944405)
Supplement: Supplementary file 1 [file Table_1.DOC]

**Supplementary table 1. Author’s judgements about study quality using the adapted Ottawa-Newcastle Risk of Bias Assessment tool**

|  | Alkhoury (2012) | Benedict (2018) | Cairo (2017) | Cheng (2018) | Gee (2021) | Halter (2016) | Gurien (2017) | Kashyap (2021) | Wakimoto (2019) | Yu (2017) | Putnam (2014) | Devin (2020) | Aguayo (2014) |
| --- | --- | --- | --- | --- | --- | --- | --- | --- | --- | --- | --- | --- | --- |
| Representativeness/appropriateness of participant selection  Random or consecutive recruitment=Y  Convenience sample=N  Not reported or unclear | Y | Y | Y | Y | Y | Y | Y | Y | Y | Y | Y | Y | Y |
| Control for baseline differences in cohorts  Similarity of groups at baseline or adjustment in analyses=Y  No attempt to control or adjust=N  Not reported=NR | Y | Y | N | Y | Y | Y | N | Y | Y | Y | Y | Y | Y |
| Loss to follow-up  Explanation provided for loss of participants and/or intention to treat=Y  No explanation =N | Y | Y | N | Y | Y | Y | Y | Y | Y | Y | Y | Y | N |
| Masking of exposure to outcomes assessor  Description of masking=Y  No masking or no description =N | Y | Y | Y | Y | Y | Y | Y | Y | Y | Y | N | Y | Y |
| Ascertainment of condition  Description of ascertainment/diagnostic criteria=Y  No description or patient self-report=N | Y | Y | Y | Y | Y | Y | Y | Y | Y | Y | Y | Y | Y |
| Documentation of other treatment modalities  Documentation=Y  No documentation=N | Y | Y | Y | N | Y | Y | Y | Y | Y | Y | Y | N | Y |
| Extent to which valid outcomes are described  Adequate description of outcome=Y  Insufficient detail regarding outcome or follow-up time=N | Y | Y | Y | Y | Y | Y | Y | Y | Y | Y | Y | Y | Y |
| Prespecification of harms, mode of harms collection  Description of a list of harms assessed or monitoring=Y  No such description or passive harms collection=N  No adverse events reported=NA | Y | Y | Y | N | N | Y | Y | N | Y | N | N | Y | N |
| Financial Conflict of interest (COI)  Funding source reported=Y  Funding source not reported=N | N | Y | Y | N | N | Y | Y | N | Y | Y | N | Y | Y |
